# Exercise Bargain: Are Walking Loops Worth the Investment?

**DOI:** 10.1289/ehp.125-A40

**Published:** 2017-02-01

**Authors:** Carrie Arnold

**Affiliations:** Carrie Arnold is a freelance science writer living in Virginia. Her work has appeared in *Scientific American*, *Discover*, *New Scientist*, *Smithsonian*, and more.

Unlike sidewalks, walking loops are designed specifically for uninterrupted exercise. In an era of shrinking budgets and expanding waistlines, preventive medicine physician Deborah Cohen of the RAND Corporation wanted to see whether walking loops are a good investment for park planners. After analyzing data for more than 170 parks, Cohen and colleagues found that those with walking loops had more users, more of whom engaged in moderate to vigorous physical activity, than those without these amenities.^1^

**Figure:**
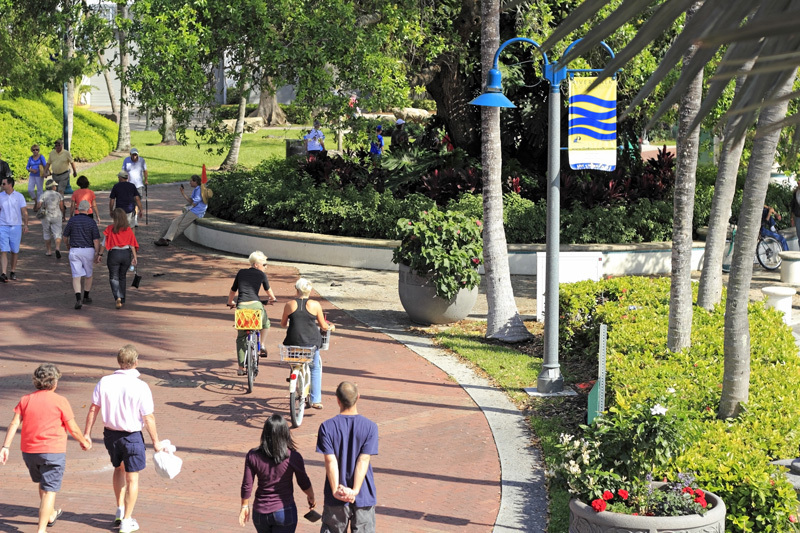
Easily navigable walking loops may be a particularly cost-effective way to help people get more exercise. © Serenethos/Shutterstock

Regular exercise is one of the best things people can do for their health. Yet in 2015 fewer than half of Americans met the federal Physical Activity Guidelines of 2.5 hours of moderate aerobic activity and 1.25 hours of vigorous aerobic activity per week.^2^ Finding a safe, accessible outdoor space is one of the major barriers to being more active, according to the American Heart Association.^3^ And fitting exercise into a busy schedule can be challenging even for people who enjoy physical exertion. “Our culture is built so that moderate to vigorous physical activity needs to be done in a person’s leisure time,” Cohen says.

Public parks can fill a needed void since they are free and do not require special skills, like joining a local sports team might. Other studies have suggested that simply being out in nature can improve both physical and mental health.^4^ Parks with walking areas may be especially important for older adults, since walking is the most popular form of exercise in this age group.^5^ Previous studies have indicated that people with access to walking loops were more likely to engage in moderate to vigorous physical activity.^6^

Parks can also foster a sense of community, especially in underserved areas, according to Jacqueline Kerr, a researcher in the division of behavioral medicine at the University of California, San Diego, who was not involved with the study. There is evidence that access to parks is restricted in less affluent areas.^7^ ^,^ ^8^

Cohen and colleagues wanted to assess how park visitors use walking loops and whether the presence of walking loops attracted people and helped them meet their activity needs. The authors used data they had gathered as part of the National Study of Neighborhood Parks. This study surveyed a representative sample of 174 parks in 25 U.S. cities with populations of more than 100,000. The parks had different combinations of amenities, such as playgrounds, athletic fields, and open green space. Fifty of the parks had walking loops.

Cohen and colleagues compared the number of park users and their levels of physical activity in parks that either did or did not have a walking loop. Study staff visited each park, making hourly observations of the number of park users, their apparent age group and gender, and their level of physical activity. In their analyses, the researchers controlled for variables including neighborhood walkability, socioeconomic indicators, and population density; the temperature on the days they observed each park; and the number of park amenities.

Walking loops were vacant only 35% of the time, whereas other park amenities were vacant an average of 75% of the time. Adults aged 20–59 were more likely to use walking loops than children or seniors, and they generally used them for walking as opposed to jogging or running. Parks with walking loops averaged 42 users per hour compared with 34 users in parks without loops. In addition, the people who used parks with walking loops tended to engage in more vigorous levels of physical activity than users of non-loop parks—even off the walking loop. The authors conclude that walking loops may be an affordable way to increase a park’s appeal and health benefits.^1^

The authors pointed out that they could not establish whether more people walked on loops because the loops were there, or whether the loops had been built in parks where people already liked to walk. They also noted it can be difficult to accurately estimate a person’s activity level, and factors such as location or aesthetic appeal could help explain why any given park is more or less popular.

Most U.S. neighborhoods are not pedestrian friendly, Kerr says, which makes Americans much less likely to walk or bike for errands than European populations. “It’s basically impossible to retrofit our environments to make them like Europe,” she concludes, “so adding in parks is a great way to make it easier for people to be active.”
